# Annotations

**Published:** 1905-08-12

**Authors:** 


### ANNOTATIONS.

### Professional Philanthropy.

In one of the current magazines attention is drawn to the existence of schemes which bear charitable and philanthropic titles but which are either absolutely fraudulent, or are so managed that the bulk of the money collected goes to provide commissions for collectors or salaries for officials. The motive of the article is to suggest that those who have money to spare for charitable purposes should take trouble to discover the true from the false, and should not allow themselves to be misled by mere loud-sounding names and pretensions. The particulars which the writer supplies fully support his contention and advice. Thus one society, which professes to meet the case of "poor children," more especially in reference to the provision of a summer holiday, shows in its last annual report the collection of a sum of over £4,000, and of this only £1,608 was expended in charity, whilst £2,270 was necessary to cover the cost of collection and management. The significance of this last item will be appreciated when it is remembered that the charges under the same head in the Children's Country Holiday Fund, which deals with an annual income of some £29,000, were less than £1,500. That is to say, whilst the Holiday Fund conducts its business at the cost of less than five per cent. of its income, the Poor Children's Society spends for a similar purpose more than fifty per cent. of its total receipts. Other associations and societies show a parallel state of affairs, and thus, avoiding altogether schemes which are fraudulent, it is obvious that the donor who wishes his money really to be used for the cause he has at heart had need scrutinise with care the business management of the charity he proposes to support. Religious professions and scriptural texts are sorry substitutes for a detailed balance-sheet and the guarantee of a professional auditor, and institutions which attempt the substitution should be scrupulously avoided.

### Babies and Board Schools.

A new departure of considerable importance has been decided upon by the Education Department of the London County Council. The Vice-President of the Council announces that school managers may henceforth be permitted to exclude from schools infants under five years of age. Better late than never. The authorities have taken many years to learn the futility of sending babies to Board schools. But futility is not the only objection. It is estimated that if the school managers in London alone avail themselves of the power given them by the Board of Education there will be a substantial saving in expenditure. This economy is desirable in the interests of the ratepayers, but by far the most serious argument against the system which has been tolerated so long is that it is absolutely injurious to the children themselves. The brains of infants under five ought not to have the strain of any kind of school curriculum imposed upon them. There is a great deal of speculation as to the reasons for the growth of insanity. We have no doubt that the development of baby intelligence at school when it should be devoted to toys and childish amusements is a contributory cause. Our only regret is that the banishment of infants from the Board School is to be optional instead of compulsory. Some managers, we are afraid, will be influenced by the cry that if infants are to be excluded from the schools their parents, who are at work during the day, will not know what to do with them. Sir William Anson anticipates that this difficulty will be met by the institution of public nurseries supported mainly by the payments of those who make use of them. We question whether people who have been in the habit of getting their babies off their hands gratuitously will be willing, even if they are able, to pay for the care of these babies while they themselves are earning money. But whatever may be the temporary barriers to the full adoption of the new departure of the Education Board, we maintain that for the common good, for the benefit of generations yet unborn, they should be swept away as soon as possible.

### Adulterated Foods.

The State Board of Health of New Hampshire issues periodically a statement of the results of the official analyses of various foodstuffs offered for sale to the public. It has the further courage to affix in each case the name and address of the manufacturer, and, as may therefore be readily imagined, the record excites considerable interest both in trading circles and among the general public. The latest published statement deals more particularly with "canned" goods, and the details are somewhat significant. Thus, of 32 specimens of fruits, jellies, and jams, only three were found to be of good quality, the balance being marked either "adulterated" or "varying from the legal standard." A comment on these facts states that the apple occupies a prominent position as the basis for many canned fruits which on their labels claim more pretentious titles. The triumphs of chemistry have placed at the disposal of the ingenious manufacturer of jam a selection of dyes and flavouring essences by which, given an adequate supply of apples, fruit jellies of almost all varieties can be produced. In this way are explained the contents of many packages claiming kinship to the strawberry, raspberry, and other fruits. The point of special interest, however, is the publication of the names of those responsible for the sale of these vamped-up products. It is, in the circumstances, hardly surprising to read that a considerable diminution of the sale of certain canned goods has followed the issue of the report. But much more welcome is the intimation that a similar policy applied at a former date to other food substances has apparently aroused the manufacturer to his obligations, as further tests in these directions have shown considerable improvement. The percentage of adulterated articles in the 363 examined was 45·2. But, as the tests are mainly concerned with substances which for various reasons have come under suspicion, these figures must by no means be applied without qualification to the sale of food products generally. At the same time, they indicate the need for constant watchfulness, and their public association with the persons responsible is a policy which might well be generally adopted.

